# Dimerization and enzymatic activity of fungal 17β-hydroxysteroid dehydrogenase from the short-chain dehydrogenase/reductase superfamily

**DOI:** 10.1186/1471-2091-6-28

**Published:** 2005-12-16

**Authors:** Katja Kristan, Dominga Deluca, Jerzy Adamski, Jure Stojan, Tea Lanišnik Rižner

**Affiliations:** 1 Institute of Biochemistry, Medical Faculty, University of Ljubljana, Vrazov trg 2, 1000 Ljubljana, Slovenia; 2 GSF-National Research Centre for Environment and Health, Institute of Experimental Genetics, Genome Analysis Center, Ingolstädter Landstraβe 1, 85764 Neuherberg, Germany

## Abstract

**Background:**

17β-hydroxysteroid dehydrogenase from the fungus *Cochliobolus lunatus *(17β-HSDcl) is a member of the short-chain dehydrogenase/reductase (SDR) superfamily. SDR proteins usually function as dimers or tetramers and 17β-HSDcl is also a homodimer under native conditions.

**Results:**

We have investigated here which secondary structure elements are involved in the dimerization of 17β-HSDcl and examined the importance of dimerization for the enzyme activity. Sequence similarity with trihydroxynaphthalene reductase from *Magnaporthe grisea *indicated that Arg129 and His111 from the αE-helices interact with the Asp121, Glu117 and Asp187 residues from the αE and αF-helices of the neighbouring subunit. The Arg129Asp and His111Leu mutations both rendered 17β-HSDcl monomeric, while the mutant 17β-HSDcl-His111Ala was dimeric. Circular dichroism spectroscopy analysis confirmed the conservation of the secondary structure in both monomers. The three mutant proteins all bound coenzyme, as shown by fluorescence quenching in the presence of NADP^+^, but both monomers showed no enzymatic activity.

**Conclusion:**

We have shown by site-directed mutagenesis and structure/function analysis that 17β-HSDcl dimerization involves the αE and αF helices of both subunits. Neighbouring subunits are connected through hydrophobic interactions, H-bonds and salt bridges involving amino acid residues His111 and Arg129. Since the substitutions of these two amino acid residues lead to inactive monomers with conserved secondary structure, we suggest dimerization is a prerequisite for catalysis. A detailed understanding of this dimerization could lead to the development of compounds that will specifically prevent dimerization, thereby serving as a new type of inhibitor.

## Background

Members of the short-chain dehydrogenase/reductase (SDR) superfamily are non-metallo enzymes with molecular masses between 25 and 35 kDa that function in the form of dimers or tetramers [1]. The crystal structures of several SDR proteins have revealed that the protein fold is highly conserved within this superfamily [2]. The SDR tetramers display 222 symmetry with two different interfaces between their 4 subunits, described by three orthogonal molecular axes, called P, Q and R [3]. The most extensive contacts are those spanning the Q-axis and involve two helices, αE and αF. In most homodimeric SDRs for which the structure is known, dimerization also occurs across this interface. So far, only one SDR protein, the 3α-hydroxysteroid dehydrogenase/carbonyl reductase (3α-HSD/CR) from *Comamonas testosteroni *(pdb code 1FK8), has been shown to differ in its mode of dimerization. In this case, dimerization takes place across the P-axis interface, formed mainly by the αG helices and by interactions between the βG strands of two neighbouring subunits [4,5]. To date, only two monomeric structures, those of porcine testicular carbonyl reductase (PTCR) (pdb code 1N5D) and human carbonyl reductase (CBR1) (pdb code 1WMA), are known within the SDR superfamily [6,7].

Although there are about three thousand members of the SDR superfamily in the species studied [8], 17β-HSD from the fungus *Cochliobolus lunatus *(17β-HSDcl) [9] is currently the only fungal HSD member that has been characterized. The enzyme has been purified, and also cloned and expressed in *E. coli *[9,10]. Under native conditions, both recombinant [10] and natural [9] 17β-HSDcl form dimers. 17β-HSDcl is homologous to fungal reductases: to versicolorin reductases from *Aspergillus parasiticus *and *Emericella nidulans*, which are involved in aflatoxin biosynthesis; and to the 1,3,8-trihydroxynaphthalene reductases and 1,3,6,8-tetrahydroxynaphthalene reductases (3HNR, 4HNR) from *Magnaporthe grisea*, *Ophiostoma floccosum *and other fungi, which are involved in melanin biosynthesis [11]. 3HNR and 4HNR catalyze an essential reaction in the biosynthesis of melanin, a virulence factor of phyto-pathogenic fungi, as well as of fungi pathogenic to humans [12-15]. These enzymes are the biochemical targets of several commercially important fungicides that are used to prevent blast disease in rice plants [15,16]. The study of the fungal 17β-HSD will, therefore, contribute to a better understanding of the functionality of these homologous fungal enzymes that are targets for the design of novel antifungal agents. As fungal 17β-HSD exhibits about 30% amino acid identity to human 17β-HSD types 4 and 8 [11], this could also lead to an understanding of the mechanisms of catalysis in human HSDs, which are implicated in the development of steroid-dependent forms of cancer, polycystic kidney disease, regulation of blood pressure, Alzheimer's disease and obesity [17-23]. A detailed understanding of the individual amino acids that are important for dimerization could enable the design of compounds that would specifically prevent dimerization, and consequently the enzyme activity of the SDR proteins, and should therefore serve as new types of drugs [24].

**Figure 1:**
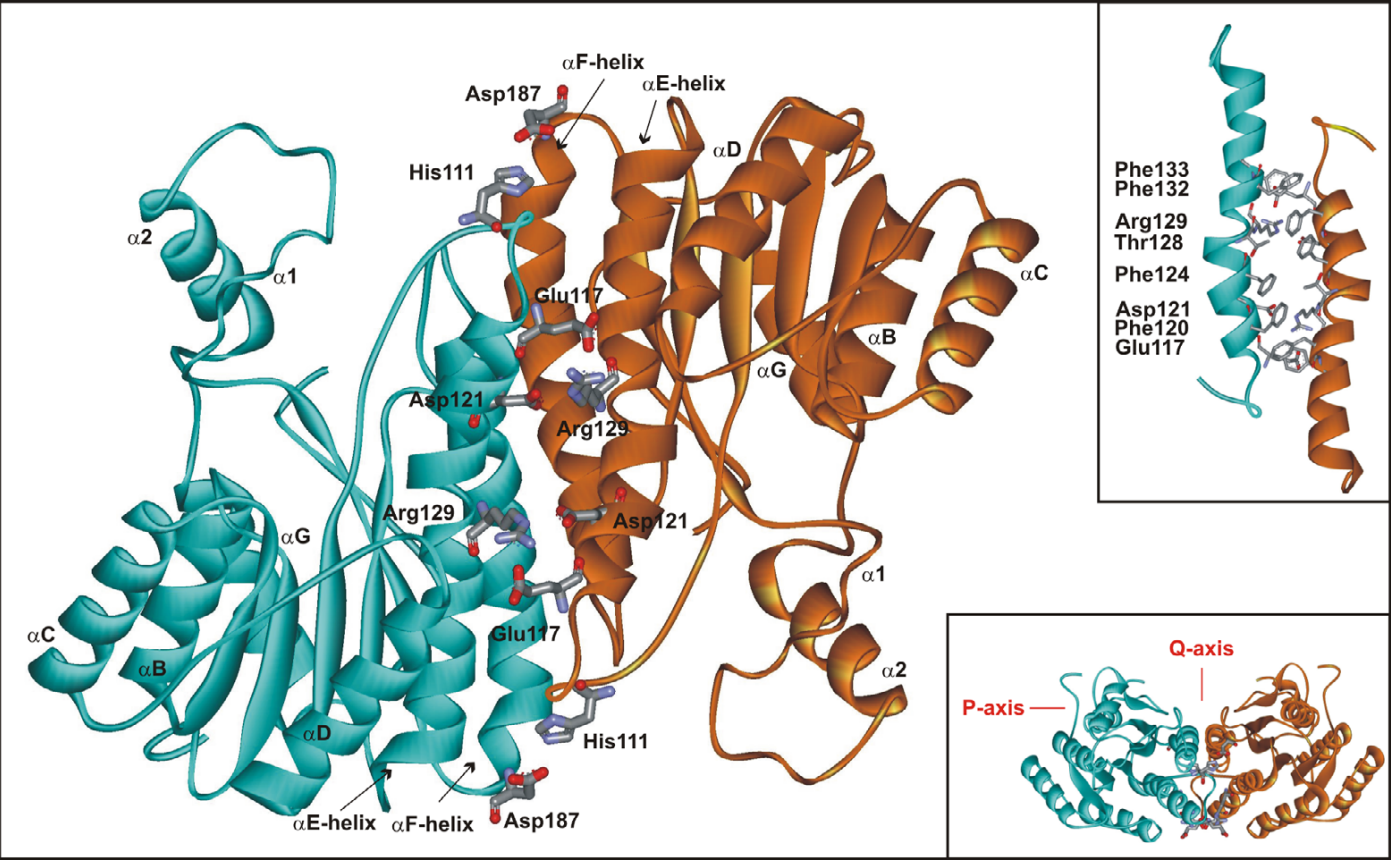
**Ribbon diagram showing the dimerization of 17β-HSDcl across the Q-axis**. The amino acids crucial for the dimerization across the Q-axes are depicted. The enlarged subunit interactions at the αE-αE contact region are also shown (inset).

Dimerization of the SDR proteins has already been studied in *Drosophila *alcohol dehydrogenase (ADH), where ethyl methanesulphonate-induced null mutants were prepared and examined. Among the mutants studied, the inactive mutant Ala159Thr did not form a stable homodimer, suggesting that Ala159 is important for hydrophobic interactions that stabilize the ADH dimer [25]. Dimerization has also been studied in human 17β-HSD type 1, an enzyme that has a high affinity for dimer formation (dimer dissociation constant *K*_*dd *_≤ 5 pM) and consequently exists only as a dimer *in vitro *[26]. The 17β-HSD type 1 mutations Leu111Glu/Val113Phe and Ala170Glu+Phe172 abolished its activity and changed the folding of the enzyme. Both mutated proteins formed aggregates with apparent molecular weights of more than 300 kDa; monomeric or dimeric forms of the enzyme were not seen [26]. Based on a comparison of the structures of fungal 17β-HSD [27,28] (Figure 1) and the homotetrameric 3HNR from *Magnaporthe grisea *(pdb code 1YBV) [12], we investigated the dimerization of 17β-HSDcl by site-directed mutagenesis. We have identified the secondary structure elements and the amino acids essential for dimerization and have demonstrated the relationship between dimerization and enzyme activity.

## Results

### Structural comparisons indicate that His111 and Arg129 are involved in dimerization

The sequence alignments and structural comparison of the three-dimensional models of fungal 17β-HSD [27], supported by our preliminary crystallographic data [28], with the homologous 3HNR [pdb code 1YBV], aligned without gaps between residue number 15 to 222 in 17β-HSDcl and residues 26 to 233 in 3HNR (60% of identical amino acids), identified strong hydrophobic and aromatic interactions across the Q-axis (Phe120, Phe124, Phe132, Phe133, Phe177) and potential salt bridges/H-bonds between the αE and αF helices of the two subunits, all of which may stabilize dimerization (Table 1, Figure 1). One salt bridge can be formed between Arg129 and Glu117 and/or Asp121 from the αE helices, while His111, which is positioned in front of the αE helix, can form an H-bond with Asp187 from the αF helix (Table 1, Figure 1). The interactions between the P-axis-related subunits are not so extensive and the surface amino acids are less hydrophobic than those of tetrameric 3HNR (Table 1). To test our hypothesis that dimerization takes place across the Q-axis, the His111Ala, His111Leu and Arg129Asp mutants were prepared with the aim of selectively omitting one H-bond (His111Ala and His111Leu) and of introducing electrostatic repulsion between the monomers (Arg129Asp).

**Table 1:** Comparisons of the amino acids potentially involved in dimerization across the Q- and P-axis interface in 3HNR and 17β-HSDcl.

**Q-axis interface**		**P-axis interface**	
**3HNR**	**17β-HSDcl**	**3HNR**	**17β-HSDcl**

*hydrophobic interactions*		*hydrophobic interactions*	
Phe131	Phe120	Phe261	Phe249
Phe135	Phe124	Trp269	Trp257
Phe143	Phe132	*salt bridges*	
Phe144	Phe133	Asp266-Arg52	
Phe188	Phe177	Asp278-Lys273	Asp266-Lys26
*salt bridges/H-bonds*			
Arg140-Asp132	Arg129-Asp121/Glu117		
His122-Asp198	His111-Asp187		

### The His111Leu and Arg129Asp mutations each result in monomeric proteins

**Figure 2:**
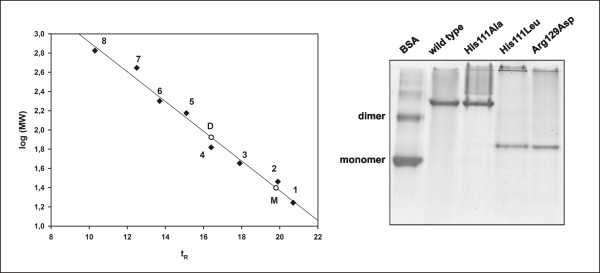
**Molecular masses**. A) Gel filtration: Native molecular masses were analyzed by gel filtration. The molecular mass standards are represented (◆) by a number from 1 to 8, respectively: myoglobin (17.5 kDa), carbonic anhydrase (29 kDa), ovalbumin (44 kDa), albumin (66 kDa), alcohol dehydrogenase (150 kDa), β-amylase (200 kDa), apoferritin (443 kDa) and thyroglobulin (669 kDa). The retention time for the dimers (D) and monomers (M) are also indicated (○). B) Native PAGE: Eight μg of the 17β-HSDcl wild-type and mutant proteins were applied to the gel, which was stained with Coomassie Blue. Bovine serum albumin (BSA) was used for comparison.

The purities of the expressed proteins were checked by SDS-PAGE, where all of the proteins appeared as bands with the expected molecular mass of 28 kDa and were approx. 90% homogenous. The solution molecular mass was determined by gel filtration and by non-denaturing PAGE. His111Ala-17β-HSDcl remained a dimer, as with the wild-type protein, while the other two mutations, His111Leu and Arg129Asp, resulted in monomers (Figure 2). The dimers eluted at the retention time of albumin with a molecular weight of 66 kDa, and the monomers eluted at a time similar to that of carbonic anhydrase (29 kDa) (Figure 2a). Differences in mobility of monomeric mutants compared to the dimeric proteins was also observed on the native PAGE (Figure 2b).

### Monomer secondary structure is conserved, although the proteins differ in their thermal denaturation characteristics

**Figure 3:**
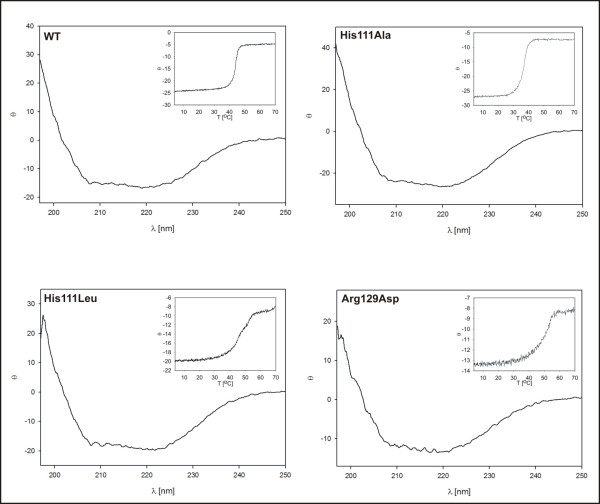
**Circular dichroism spectra and thermal denaturation**. The circular dichroism spectra were recorded by measuring the ellipticity as a function of wavelength at 0.1 nm increments between 260 and 197 nm at 20°C. The thermal denaturation was determined by measuring the ellipticity as a function of temperature at 0.1°C increments between 4 and 70°C (inset). The protein concentrations used were approximately 0.2 mg/ml.

The secondary structures of the wild-type and mutant enzymes were analysed through circular dichroism spectroscopy. This method revealed similar conformations for all the proteins (Figure 3). The thermal denaturation was measured by following the change of the ellipticity at 222 nm as a function of the temperature. Two behaviours were recognized, that of the wild-type protein and the His111Ala mutant, and that of the two monomeric mutants, His111Leu and Arg129Asp. The melting curves of the wild-type protein and the His111Ala mutant were sigmoidal, revealing a cooperative denaturation. The wild-type protein had a T_m _of 44°C, and the His111Ala mutant, of 37°C. The forms of the melting curves of the other two mutants suggested lower cooperativities of denaturation. The melting points were 48°C for the His111Leu mutant and 50°C for Arg129Asp (Figure 3) (Table 2).

**Table 2:** Kinetic constants and T_m _values for the wild-type and mutant 17β-HSDcl

	*K_*d*_^*NADP*+ ^(μM)	*K*_*M*_^*NADPH *^(μM)	*K*_*M*_^*NADP*+ ^(μM)	*k*_*cat*_^*NADPH *^(s^-1^)	*k*_*cat*_^*NADP*+ ^(s^-1^)	*k*_*cat*_/*K*_*M*, *NADPH *_(s^-1^M^-1^)	*k*_*cat*_/*K*_*M*, *NADP*+ _(s^-1^M^-1^)	T_m _(°C)
*Wild type*	0.2	6.5	0.06	3.8	0.65	5.8 × 10^5^	1.1 × 10^7^	44
*His111Ala*	5.8	50.6	4.6	2.4	0.9	4.7 × 10^4^	1.9 × 10^5^	37
*His111Leu*	19.9	N.A.	N.A.	N.A.	N.A.	N.A.	N.A.	48
*Arg129Asp*	12.8	N.A.	N.A.	N.A.	N.A.	N.A.	N.A.	50

Kinetic constants for the reduction and oxidation of the substrates 4-estrene-3,17-dione and 4-estrene-17β-ol-3-one (100 μM) (*K*_eq _= 2.46 ± 0.11 [38]) that were catalyzed by the wild-type and mutant 17β-HSDcl in the presence of the coenzymes NADPH and NADP^+ ^(100 μM), at pH 8 and 25°C. The temperature midpoints (T_m_) are also given. (* determined by fluorescence titrations; N.A.: no activity detected)

### The coenzyme binds to the monomeric mutants

We measured the formation of the binary complexes of E•NADP^+ ^for the wild-type and mutant enzymes. The fluorescence of all of the proteins was quenched by the addition of the coenzyme, but the *K*_*d *_values differed. The *K*_*d *_value for the dimeric mutant His111Ala increased 30-fold, and those for the His111Leu and Arg129Asp monomers increased 100-fold and 60-fold, respectively, when compared with the wild-type enzyme (Table 2). These results support the circular dichroism data, suggesting only minor changes in the coenzyme binding site, even in the monomeric enzymes.

### The His111Leu and Arg129Asp mutants are not active on the substrates

The oxidation of 4-estrene-17β-ol-3-one and the reduction of 4-estrene-3,17-dione by the wild-type and mutant proteins in the presence of NADP^+ ^and NADPH showed enzyme activities for the wild-type protein and for the His111Ala mutant, but no detectable activities for the monomeric His111Leu and Arg129Asp proteins within a 450 s sweep. Although the His111Ala mutant was active, its kinetic constants differed from those of the wild-type protein (Table 2). The *K*_*M *_values for both of the coenzymes, NADP^+ ^and NADPH, were increased in the His111Ala mutant (77-fold and 8-fold, respectively), while the *k*_*cat *_values remained similar (Table 2). The specificity constants, *k*_*cat*_/*K*_*m*_, for NADP^+ ^and NADPH, were 58-fold and 12-fold lower, respectively, when compared to those for the wild-type enzyme (Table 2).

Since we could not detect any enzyme activities with the monomeric mutants within the 450 s sweep, we also tested the enzyme activities by TLC analysis of the products after 24-h incubations of the proteins with 4-estrene-17β-ol-3-one and NADP^+^. Again, the wild-type and His111Ala mutant proteins converted this substrate to equilibrium concentrations, while the His111Leu and Arg129Asp mutant proteins did not (data not shown).

## Discussion

### The dimerization of fungal 17β-HSD occurs across the Q-axis

Among the tens of SDR enzymes for which the three-dimensional structures have been resolved, only two are known to be active in solution as monomers (pdb code 1N5D and 1WMA) [6,7]. We have shown previously, that the wild-type 17β-HSDcl acts as dimer [10]. In order to influence the formation of this dimer, we substituted two surface amino acids which should not, *per se*, prevent either the folding or the activity. To avoid possible cooperative phenomena, we prepared mutants with single substitutions, yet we knew that in the dimer the effects would be doubled.

Inspection of the three-dimensional structural model of 17β-HSDcl [27] supported by our preliminary crystallographic data [28] revealed that His111 from each subunit could form an H-bond with Asp187 from another subunit, while Arg129 could form salt bridge(s) with two negatively charged candidates, Asp121 and Glu117. The importance of these interactions was confirmed directly with three mutations: His111Ala/Leu and Arg129Asp. The His111Ala mutation resulted in dimers, while that of His111Leu prevented the formation of an H-bond as well as the formation of a dimer, most probably because of the steric hindrance introduced by Leu (Figure 2). Substitution of Arg129 by Asp results in electrostatic repulsion between the monomers, and simple calculations revealed that the two abolished salt-bridges result in an approximately 10^14^-fold higher dimer dissociation constant (*K*_*dd*_), thus preventing dimerization. On the other hand, if all of the binding energy were realized in the affinity change caused by two H-bonds, as in the His111 mutants, only a half of the above change would be expected. It appears that this is still not enough to prevent dimerization (as with His111Ala), unless the steric component is added (as with His111Leu). Since both of the substituted residues are the members of the αE helix, these results strongly suggest that dimerization of fungal 17β-HSD takes place across the Q-axis.

### The denaturation of monomers occurs at higher temperatures

The results showed that the thermal denaturation of the wild-type protein and the dimeric His111Ala mutant is a cooperative process that involves the disruption of the dimers and the denaturation of the monomers. Compared with the wild-type protein, denaturation of this mutant occurred at a lower temperature (Table 2). The results indicated that the mutation introduced causes a decrease in the thermal stability of the protein, and that the His in position 111 is involved in interactions that stabilize the dimer.

The higher melting temperature of the two monomeric mutants, His111Leu and Arg129Asp, may be associated with a higher stability of the monomers and with a decreased flexibility of their structure in comparison with the dimers. A higher flexibility may be needed to rearrange the conformation after binding of the cofactor, thus enabling proper binding of the substrate, and this may also explain the loss of activity of the His111Leu and Arg129Asp mutants. Additionally, the icebergs of solvent (water) around the exposed hydrophobic groups of the αE and αF helices may contribute to the higher Tm. As the temperature is raised, the icebergs melt and their latent heat of fusion contributes significantly to the specific heat capacity (*Cp*) of the denatured state [29].

### Dimerization is crucial for enzyme activity

The substitution of His111 by Ala at the Q-interface resulted in higher *K*_*d *_values and lower specificity constants (*k*_*cat*_/*K*_*M*_) of this dimer when compared with the wild-type enzyme, while the substitution of His111 by Leu and of Arg129 by Asp resulted in inactive monomers. These results indicate that H-bonds/salt bridges contribute to both the specific dimerization and the enzyme activity. The stability of the Q-interface, which is composed of the αE and αF helices, is crucial for enzyme catalysis in the SDR enzymes since two catalytic residues, Tyr(167) and Lys(171), reside in the interior side of the αF helix, one catalytic residue, Asn(127), resides in the interior side of the αE helix, while Ser(153) is positioned in the small αEF helix, between the βE strand and the αF helix [1,30]. The proper orientation of these catalytically important helices assures the proper orientation (setting, proximity, distance) of the catalytic amino acids to the nicotinamide ring and the substrate, enabling the hydride transfer.

The investigation of another SDR member, glucose dehydrogenase (GlcDH) from *Bacillus megaterium *IWG3 (pdb code 1RWB) [31], supports our hypothesis. GlcDH functions as a tetramer and its crystal structure has revealed weaker hydrophobic interactions and a lack of salt bridges in the Q-interface. For these reasons, the GlcDH tetramer dissociates into inactive monomers at pH 9.0, but reversibly associates into the fully active tetramer at pH 6.5. This is not the case for our fungal enzyme, which is active in the pH range of 7.0 to 9.0 [32]. Our results and those of others have thus demonstrated that only upon dimerization can the key catalytic residues be brought into the positions that enable the substrate to be converted.

Since both of the mutant monomers show no enzymatic activity despite their conserved secondary structures and the positive coenzyme binding (Table 2), it appears that the enzyme-coenzyme (E•NADP(H)) binary complex undergoes conformational changes before the substrate binding can occur. Such a conformational adaptation has previously been proposed for 3α-HSD from the SDR superfamily and for several members of the aldo-keto reductase superfamily [33,34]. In these proteins, the binding of NADP(H) involves two events: an initial formation of a loose complex (E•NADP(H)), followed by a conformational change in the enzyme structure that leads to a tightly bound complex (E*•NADP(H)) [33,34]. Our data are in agreement with his hypothesis.

### Dimerization via the Q-axis is not possible in 3α-HSD/CR, PTCR and CBR1

On comparing the crystal structures of dimeric and tetrameric SDRs with those of 3αHSD/CR, where dimerization takes place via a P-axis interface [4,5], and of PTCR and CBR1, the only monomeric enzyme structures of the SDR superfamily [6,7], it becomes evident that in all three structures, interactions across the Q-axis are blocked by the presence of a predominantly α-helical subdomain. In these three proteins, this 28, 41 or 41 amino-acid insertion into the classical Rossman-fold motif between strand βE and helix αF, respectively, prevents the formation of a four-helix bundle, while at the same time it masks, and in this manner stabilizes, the αE and αF helices. To determine whether the lack of this subdomain leads to the formation of a homotetramer, a redesign of this interface was recently performed in the dimeric 3α-HSD/CR, and further enzymatic characterization is currently in progress [34]. This insertion is not present in other SDRs that can stabilize the αE and αF helices by dimerization via the Q-axis interface. Therefore, again, dimerization appears to be crucial for the stability of the αE and αF helices in the fungal 17β-HSD as well as in other SDR proteins, except 3α-HSD/CR, PTCR and CBR1.

### Selective prevention of dimerization may enable the development of a new type of drug

A detailed understanding of the importance of specific residues for dimerization should enable the design of compounds that can prevent the dimerization and thus serve as a new type of drug. This proposal is supported by the finding that the residues involved in subunit interactions differ considerably between the enzymes. Recently, Caffrey et al. (2004) examined the difference in conservation between the protein-protein interfaces and the rest of the protein surfaces for a set of 64 proteins from different protein superfamilies [36]. A surface-patch analysis showed that this interface is rarely significantly more conserved than other surface patches [36]. Although almost all of the SDR proteins span across the Q-axis interface, the residues involved in the dimerization are not conserved. Subunit interactions at the αE-αE contact regions were compared in 6 members of the SDR superfamily (GlcDH, mouse lung carbonyl reductase, *Streptomyces hydrogenans *3α,20β-HSD, *Pseudomonas *sp. *cis*-biphenyl-2,3-dihydrodiol-2,3-dehydrogenase, *Escherichia coli *7α-HSD, *Magnaporthe grisea *3HNR) [31], whereby different modes of subunit interactions were shown in these proteins. Therefore, the concept that the selective prevention of dimerization in the SDR proteins will lead to the development of a new type of inhibitors is reasonable.

## Conclusion

Fungal 17β-HSD is a dimeric member of the SDR superfamily. We have shown by site-directed mutagenesis and structure/function analysis that the dimerization takes place across the Q-axis and involves the αE and αF helices of both subunits. Neighbouring subunits are connected through hydrophobic interactions, H-bonds and salt bridges involving amino-acid residues His111 and Arg129. The substitution of these amino acid residues leads to inactive monomers with conserved secondary structures that are still able to bind the coenzyme. Our results indicate that the stability of the αE and αF helices that include catalytic amino acid residues is crucial for enzyme activity. Although oligomerization of the SDR proteins has already been studied in *Drosophila *alcohol dehydrogenase and human 17β-HSD type 1, our study is the first that has prepared monomeric mutants in sufficient quantities and qualities for structure/function analysis.

## Methods

### Preparation of mutant proteins

The mutant proteins were prepared using the Quick-Change Site-Directed Mutagenesis Kit (Stratagene) and the pGex-17β-HSDcl expression vector [10]. The following primers were used (only mutagenic forward primers are shown; the mutations introduced are underlined):

Arg129Asp

for: 5'-GCCTCAACACCGACGGCCAGTTCTTCG-3'

His111Ala

for: 5'-CGTGAGCTTCGGCGCCCTCAAGGACGTG-3'

His111Leu

for: 5'-CGTGAGCTTCGGCCTCCTCAAGGACGTG-3'

The fidelity of the constructs was confirmed by dideoxy sequencing.

### Expression and purification of proteins

All of the proteins were expressed as GST-fusion proteins in the JM107 *E. coli *strain and purified by affinity chromatography on Glutathione Sepharose followed by cleavage with thrombin, as described previously [10].

### SDS PAGE

Homogeneity of the proteins was checked by SDS-PAGE. Samples (4 μg) were denatured in Laemmli sample buffer (5 min at 90°C), applied to 12% acrylamide gels, and visualized by Coomassie Blue staining.

### Estimations of molecular mass

#### Gel filtration

The molecular masses were determined by gel filtration on BioCad Sprint FPLC. The mobile phase was 100 mM phosphate buffer (Na_2_HPO_4_/NaH_2_PO_4_) with 150 mM NaCl, pH 7.0. Approximately 40 μg of protein in 100 μl was injected onto a TSK G4000 SWxl column (7.5 mm ID × 60 cm) and analyzed at a 1.0 ml/min flow rate. Elution of the proteins was followed at 280 nm. The void volume of the column was determined with blue dextran. The calibration standards were: myoglobin (horse heart) (17.5 kDa), carbonic anhydrase (bovine erythrocyte) (29 kDa), ovalbumin (albumin from hen egg white) (44 kDa), albumin (bovine serum) (66 kDa), alcohol deydrogenase (yeast) (150 kDa), β-amylase (sweet potato) (200 kDa), apoferritin (horse spleen) (443 kDa) and thyroglobulin (bovine) (669 kDa).

#### Non-denaturing PAGE

Eight μg of protein were run on 9% acrylamide gels, as described previously [37]. Following electrophoresis at 150 V, the proteins were stained by Coomassie Blue.

### Secondary structure determinations

Preservation of the secondary structures was followed by circular dichroism spectroscopy (JASCO Corp. J-715 spectropolarimeter; PFD-350S temperature controller; maintained by J-700 software package). The spectra were recorded at 20°C at 0.1 nm increments between 250 and 197 nm using 0.1 cm path length cuvettes. The scanning speed was 50 nm/min, the response time 1 sec, and an average of 4 scans was recorded. The protein concentrations were approximately 0.2 mg/ml, in PBS (pH 7.3).

### Thermal denaturation of proteins

Ellipticity was measured at 222 nm at 0.1°C increments between 4 and 70°C. The temperature was raised at 30°C/h, the response time was 16 sec, and the bandwidth was 1 nm. At the end of each experiment the temperature was decreased to 4°C to verify the reversibility of the denaturation.

### Coenzyme binding

To determine coenzyme binding constants, we measured the changes in the intrinsic enzyme fluorescence upon the incremental addition of coenzymes (0–30 μM) on a Cary Eclipse fluorescence spectrophotometer (Varian). To ensure that the volume of coenzyme added was not more than 3%, three stock solutions of the coenzyme were prepared – 15 mM, 1.5 mM and 150 μM. A 4 × 10-mm cuvette with a magnetic stirrer was used, and each 800 μl sample contained 1 μM protein in 100 mM phosphate buffer, pH 8.0 at 25°C. The samples were excitated at 290 nm with the fluorescence emission scanned at 335 nm and with the excitation and emission band-passes set at 5 nm. Single or double hyperbolic equations were fitted to the data to estimate the corresponding dissociation constants for the wild-type and mutant forms of 17β-HSDcl.

### Enzyme activities

The interconversion between 4-estrene-3,17-dione and 4-estrene-17β-ol-3-one in the presence of NADPH or NADP^+ ^catalyzed by the wild-type and mutated enzymes was followed photometrically at 340 nm, in 100 mM phosphate buffer, pH 8.0 at 25°C. In all of the experiments, 1% DMF was present, to enhance substrate solubility. The time course of absorbance at 340 nm was measured for 450 s. The progress curves were analyzed according to the procedure described previously [38].

#### Thin-layer chromatography (TLC)

The wild-type and mutant proteins (0.5 μM) were incubated with 4-estrene-17β-ol-3-one (100 μM) in the presence of NADP^+ ^(500 μM) in 100 mM phosphate buffer, pH 8.0, for 24 h at room temperature. The steroids were extracted with chloroform and analyzed by TLC as described previously [37].

## List of abbreviations

17β-HSDcl, 17β-hydroxysteroid dehydrogenase from *C. lunatus*; 3HNR, 1,3,8-trihydroxynaphthalene reductase; 4HNR, 1,3,6,8-tetrahydroxynaphthalene reductase; CBR1, human carbonyl reductase; CR, carbonyl reductase; GlcDH, glucose dehydrogenase; GST, glutathione S-transferase; HSD, hydroxysteroid dehydrogenase; PTCR, porcine testicular carbonyl reductase; SDR, short-chain dehydrogenase/reductase

## Authors' contributions

KK carried out the bulk of the experimental work presented herein (site-directed mutagenesis, enzyme purifications, fluorescence measurements, enzymatic assays). DD was integral to the secondary structure determinations of the enzymes, and the thermal denaturation and gel filtration. JS analyzed the progress and titration curves, determined the rates and kinetic constants, and participated in the interpretation of the results. TLR and JA participated in the conception of the study, in the design of the study, and in the writing and revising of the manuscript. All of the authors have read and approved the final manuscript.
